# Reliability and Repeatability of a Postural Control Test for Preterm Infants

**DOI:** 10.3390/ijerph20031868

**Published:** 2023-01-19

**Authors:** Katarzyna Kniaziew-Gomoluch, Andrzej Szopa, Tomasz Łosień, Jan Siwiec, Zenon Kidoń, Małgorzata Domagalska-Szopa

**Affiliations:** 1 Department of Physiotherapy, School of Health Sciences in Katowice, Medical University of Silesia, 40-752 Katowice, Poland; 2 Rehabilitation and Medical Center Neuromed in Katowice, 40-698 Katowice, Poland; 3 Department of Developmental Age Physiotherapy, Medical University of Silesia, 40-752 Katowice, Poland; 4 John Paul II Pediatric Center, 41-218 Sosnowiec, Poland; 5 Department of Electronics, Electrical Engineering and Microelectronics, Silesian University of Technology, 44-100 Gliwice, Poland

**Keywords:** pre-term infants, General Movements Assessment, postural control, center of preassure displacement

## Abstract

Background: the current study aims to evaluate the reliability and repeatability of a new PT based on Center of Pressure (CoP) movement analysis in a repeated measures design. Methods: the examination consisted of two parts: (1) the videotaping of General Movements (GMs) and GMs assessment (GMA) and (2) Posturometric Tests (PT) in supine and prone positions. PTs were performed twice (by two investigators) in the supine and prone positions using a force plate. Based on the GMA results, infants were stratified into two groups: (1) infants with normal FMs (indicating normal future motor outcomes) (*n* = 18) and (2) infants with abnormal FMs (indicating later neurological dysfunction) (*n* = 19). Results: the comparative analysis between the groups of infants with normal FMs and abnormal FMs in PT in supine showed significant differences for all parameters that described spontaneous CoP displacement. The reliability analysis determined that all ICCs of the outcomes presented at least a moderate level of reliability. The ICCs were higher for outcomes of PT performed in the supine position than in the prone position. The ICCs were higher for outcomes of PT performed in infants with abnormal vs. normal FMs. Conclusions: although the current study yielded promising results, further longitudinal research in preterm infants should identify whether altered postural control parameters prognose future motor outcomes.

## 1. Introduction

The early diagnosis of neurodevelopmental impairment (NDI), including cerebral palsy (CP), remains a challenge for both clinicians and researchers. The general opinion is that the identification of early symptoms (up to 3 months old) of NDI development, including CP, in premature infants is extremely challenging [1]. A review of the results of studies on the prognostic value of diagnostic tools for the recognition and prognosis of NDI in newborns and infants who were <6 months of age indicated that most of the tools or scales used for the assessment of psychomotor development of premature infants remain unclear [2]. These findings showed that the tools with the highest predictive validity for infants up to 5 months of age (corrected age) are: (1) neuroimaging with the use of magnetic resonance imaging (MRI, sensitivity 86–89%); (2) Prechtl’s General Movements Assessment (GMA, sensitivity 98%); and Hammersmith Infant Neurological Examination (HINE, sensitivity 90%). It is currently accepted that the most evidence-based clinical approach for the prediction of motor impairment in preterm infants is clinical assessment based on the analysis of quality of general movements [1,3]. The presence of normal GMs in a child’s motor development, including (1) writing movements (WM) present from 40 weeks of gestational age to week 9 post-term and (2) fidgety movements (FMs) present from week 9 to week 20 post-term, are strong predictors of neurologically normal psychomotor development [4,5]. The presence of abnormal GMs patterns during preterm and term age include: (1) poor repertoire GMs (PR); (2) cramped-synchronized GMs (CS); (3) chaotic GMs (CA) [4,5]; and (4) absent FMs and abnormal FMs in the post-term postmenstrual age (at 9–12 weeks), and they indicate an increased risk for later neurological dysfunction [6,7]. Absent FMs at 3 to 5 months after term (instead of normal FMs) are particular early markers of adverse neurological outcomes [6,7]. Although, an analysis of the quality of GMs allows (with high reliability, sensitivity, and specificity) to predict the development of NDI, including CP, it is based on subjective visual assessment [4,5,6,7].

With advances in computer science, multiple systems based on a motion capture system or body-worn miniaturized movement sensors have been adopted to identify general movement (GM) patterns in young infants [8]. Adde et al. carried out a series of studies based on a video analysis to identify quantitative movement differences between different types of GMs and used them for the prediction of CP in preterm infants [9,10,11]. One limitation of this analysis is that it mainly reflects the spatial characteristics and does not include enough specific temporal features of infants’ movements [8]. Støen and colleagues analyzed the temporal organization of infants’ movements and found significant differences between several categories of GMs, such as normal FMs, absent FMs, and abnormal FMs [12]. Although the results of these studies seemed promising, none were sensitive enough to determine movement parameters that could distinguish normal GMs from abnormal GMs [8,9,10,11]. Moreover, as shown by the results of a review of “automated movement recognition technologies to assess infant movement” performed by Marcroft et al., the applicability of these techniques is limited by difficulties attaching special markers to the very small limbs of infants [13].

The Center of Pressure (CoP) methodology with pressure-sensitive mats has been used for some time for infant postural and motor control assessment [14,15]. Although, the results of the CoP displacement were sufficient to stratify infants by birth status (preterm/term), and these studies did not target the population of infants at high risk for developing NDI. CoP methodology has been also used in novel devices based on integrated computer-based video systems and sensor supported systems, such as Play and Neuro-Development Assessment (PANDA) [16] or Care Toy [17]. PANDA is an integrated sensor system combination that includes GoPro cameras, a set of toys with sensors, and a mat structure, and it measures the displacement of CoP in infants [16]. Care Toy is a modular device that includes inertial and magnetic measurement units, sensorized toys, and pressure-sensitive mats (PSM) [17]. The connecting element of both PANDA and Care Toy is the registration of CoP displacement from PSM while infants lay supine.

While the results of several studies comparing various CoP indices from PSM in combination with movement analysis systems between infants grouped by future motor outcome (typical motor control/impaired motor control) [16,17,18] seemed very promising, they did not refer to any clinical classification of infants at high risk of developing NDI according to GMA.

Early validation of CoP displacement in preterm infants (up to 3 months of age), grouped by GMA outcomes (the presence of normal FMs/absent FMs) measured using a force platform in a horizontal position (supine or prone) was not assessed until we published our last study [19,20]. In this study, we characterized a new posturometric test (PT) based on CoP movement analysis in terms of design and construct validity for the detection of postural control disturbances in preterm infants [19,20]. Based on the results, we concluded that a new PT in a supine position was valid for the stratification of children who presented with normal and abnormal FMs [19].

Thus, the current study aims to evaluate the reliability and repeatability of a new PT based on CoP movement analysis in a repeated measures design. We hypothesized that postural control parameters that describe CoP displacement registered by new PT in the horizontal positions would allow us to distinguish infants with normal FMs and abnormal FMs. We also hypothesized that PT would display better reliability and test–retest repeatability in the group of infants with abnormal FMs than in the group of infants with normal FMs.

## 2. Materials and Methods

This study protocol was approved by the local Bioethical Committee under resolution No. KNW/0022/KB1/148/14.

### 2.1. Participants

Forty pre-term infants who were cared for by the local Neonatal Counsel Clinic of the Public Clinical Hospital and qualified for the SYNAGIS program (prophylactic respiratory syncytial virus infection) were enrolled in the study. These infants participated in our previous research project on the characterization of the present posturometric test in terms of design and construct validity for the detection of postural control disturbances in preterm infants [19,20].

The inclusion criteria for both groups were as follows: (1) infants’ gestational age at birth was between 24 and 33 completed weeks; (2) the infants were clinically stable; and (3) legal guardians’ provided approval for the examination. Participation was excluded if (1) they had major congenital anomalies and genetic syndromes or (2) they had an infection or inflammation during the examination.

The study population consisted of two groups of pre-term infants. The study group included 19 infants (6 girls and 13 boys) who presented with brain ultrasound abnormalities and abnormal FMs at 12–14 weeks after term. The control group enrolled 18 individuals with normal brain ultrasound findings and FMs at 12–14 weeks after term who were matched for sex and age (in a 1:1 case-control manner) to the children from the study group. Characteristics of both groups of infants, i.e., the group of infants with normal FMs and the group of infants with abnormal FMs, are presented in Table 1. All parents or caregivers gave their informed consent prior to participating in the study.

### 2.2. Examinations

The examination consisted of (1) the videotaping of GMs and GMA and (2) PT in supine and prone position.

Assessment of GMs consisted of capturing spontaneous activity on video at 12–14 weeks after the infant’s due date, according to the standard methodological principles of Prechtl’s Method [4,5,21]. Three representative examples of GMs from the one-day activity of an infant were copied and recorded as one video clip. Based on those video clips, two independent observers certified by Prechtl’s Method scored GMs as normal FMs or absent FMs. Based on GMA results, infants were stratified into two groups, including (1) infants with normal FMs and (2) infants with abnormal FMs.

**Figure 1:**
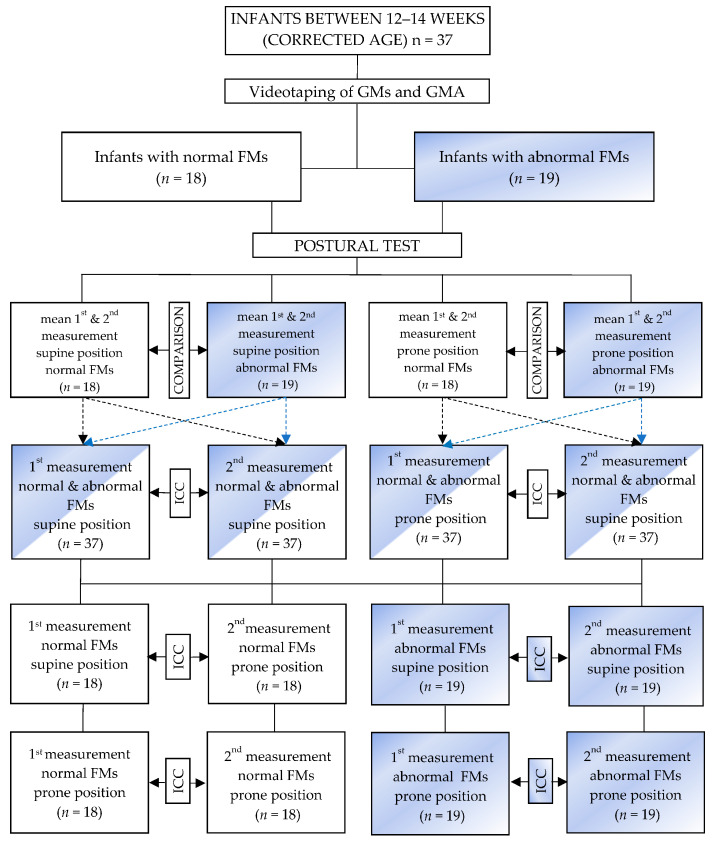
Study design.

The study protocol we followed in this study was mostly the same protocol we used in our previous study [19]. Posturometric tests was performed twice and simultaneously with GMs recording in both positions—first in supine, and then in prone. A force plate with dedicated software and a video recorder connected to the computer (a device designed and manufactured in the Department of Biomedical Electronics of the Institute of Electronics of the Silesian University of Technology in Gliwice) were used for the PT [19]. The differences between this study’s protocol and our previous study’s [19] is that PT was conducted twice on the same day in each position in this study, including once by investigator number one and once by investigator number two. The interval between the examinations was not less than 6 h. In addition, much more posturometric parameters were analyzed than in the previous study (Table 1). The study design is presented in Figure 1.

The postural indices and the formulas used to calculate them are presented in Table 2 [22].

### 2.3. Statistical Analysis

The software package SPSS v26.0 (IBM Corp., Armonk, NY, USA) was used to perform all statistical analyses. Sensitivity analyses for *t*-tests for two independent groups were performed using G*Power 3.1.9.4 [23,24]. This showed that with a sample size consisting of *n*1 = 19 and *n*2 = 18 participants, α = 0.05, power = 0.80, and the required minimum effect size for a two-tailed analysis was Cohen’s d = 0.92.

The normality of the quantitative variables was tested using Shapiro–Wilk tests (*p* > 0.05). Frequencies, percentages, means (with standard deviations), or medians (ranges) as appropriate were used to describe quantitative and qualitative variables. Since all data were found to be in line with a normal distribution or slightly different from a normal distribution, with the value of skewness ranging from −1 to 1 and the value of kurtosis ranging from −3 to 3, the statistically significant differences in the posturometric parameters between the groups were determined using the student’s *t*-test for independent variables. All the posturometric measurements were determined twice for the comparison of posturometric parameters between the groups, which used an average measurement index for each parameter from the first and second measurements. All the posturometric measurements were made in supine and prone positions, and all analyses were performed separately for each position. For both measures (in supine and in prone), an intraclass correlation coefficient (ICC) analysis was performed between the first and second measurements [25]. All results were considered significant at *p* < 0.05.

The Pearson Correlation test was used to examine the relationship between the outcomes of PT in the supine and prone positions. The correlations were performed separately for the group of infants with normal FMs and the group of infants with abnormal FMs. The correlations were interpreted according to the guidelines adopted from Altman [26], which include the following: *r* < 0.30, negligible correlation; 0.31–0.50, low positive (negative) correlation; 0.51–0.70, moderate positive (negative) correlation; 0.71–0.90, high positive (negative) correlation, and 0.91–1.00 very high positive (negative) correlation. Coefficients with a *p*-value of less than 0.05 were considered significant.

## 3. Results

Differences in CoP parameters describing spontaneous sway of CoP and the area of CoP between the groups of infants with normal FMs and infants with abnormal FMs in PT in supine and prone positions are shown in Table 3 and Table 4.

The comparative analysis between the groups of infants with normal FMs versus abnormal FMs in PT in the supine position showed significant differences for all parameters describing spontaneous CoP displacement in the supine position (Table 3). They concerned both posturometric indices based on CoP shifts, i.e., the velocity of the CoP displacement (Vmean CoP and VmaxCoP) and the sway path of the CoP (SPL) and the linear displacement of the CoP in left, right, up, and down (MaxCoPR, MaxCoPL, MaxCoPup, and MaxCoPdown), as well as posturometric indices based on the surface area of the CoP (ACoP, MDevCoP, MCoPx, MCoPy, and AR) (Table 3).

Results regarding PT in the prone position showed that only posturometric indices based on the surface area of the CoP (ACoP, MDevCoP, MCoPx, and MCoPy) distinguished infants with abnormal FMs from those with normal FMs (Table 4). There were no statistically significant differences in postural indices based on CoP shifts and displacement (Vmean CoP, VmaxCoP, SPL, MaxCoPR, MaxCoPL, MaxCoPup, and MaxCoPdown) found between the group of infants with normal and abnormal FMs (Table 4).

Subsequent steps of the analysis were performed on the basis of separate outcomes of two PT measurements in both positions (supine and prone), taken on in the same day approximately 6 h apart. 

There were no differences detected between any postural parameters on both measurements for the whole group as well for both subgroups (all parameters had a *p* > 0.05). Then, the relative test–retest reliability based on individual parameters describing the spontaneous sway of CoP and the area of CoP between two measurements in both positions (supine and prone) in the whole group of infants (Table 5) and both subgroups of infants (normal FMs and abnormal FMs) (Table 6) was determined using the ICC for test–retest data [27]. ICC values less than 0.5 were considered to have poor reliability, while values between 0.5 and 0.75 were considered to have moderate reliability, values between 0.76 and 0.9 were considered to have good reliability, and values greater than 0.90 indicated excellent reliability [27].

Overall, the reliability analysis performed on the whole group of infants determined that the ICCs were higher for outcomes of PT performed in the supine position than in the prone position. (Table 5). All ICCs of outcomes presented at least a moderate level of reliability (ICCs above 0.5), while the rest of the ICCs indicated good and excellent reliability (Table 5). The highest values of repeatability were obtained for posturometric indices based on the surface area of CoP. ACoP and MDevCoP had excellent reliability (CCI > 0.9) and MCoPx and MCoPy indicated good reliability (CCIs between 0.76 and 0.9). Postural indices based on CoP shifts (Vmean CoP and SPL) and CoP displacement (MaxCoPR, MaxCoPL, MaxCoPup, and MaxCoPdown) presented moderate reliability (ICC between 0.76 and 0.9) (Table 5). A similar trend concerns the outcomes from measurements in the prone position; however, the ICC values were lower (Table 5).

The reliability analysis performed separately in the subgroups of infants determined that the ICC values were less variable in infants with abnormal FMs and indicated a higher level of reliability compared with children with abnormal FMs patterns (Table 6). Specifically, the ICCs of the abnormal FMs group ranged from 0.52 to 0.97 and presented only good and excellent reliability, and in particular, this concerned the following posturometric indices based on the surface area of CoP: ACoP and MDevCoP in the supine position (Table 6).

At the end of the analysis, the correlations between the outcomes of PT in supine and prone positions were checked (Table 7). The aim of the analysis was to see if there were differences between infants with normal versus abnormal FMs regarding the strength of correlations between PT in supine and prone positions. Statistically significant correlations were found only in infants who presented with abnormal FMs, while no significant correlations were observed between parameters measured in supine and prone positions in infants who presented with normal FMs (all parameters *p* > 0.05). Pearson’s correlation tests found a high positive correlation between the variables of posturometric indices based on the surface area of the CoP, including ACoP, MDevCoP, MCoPx, and MCoPy (r between 0.69 and 0.73). Additionally, a high and moderate positive correlation was found between the variables of posturometric indices based on CoP shifts and displacement (SPL and Vmean CoP).

## 4. Discussion

A new PT based on the displacement of the CoP on the base of support (BoS) measurement during the infant’s maintenance of supine and prone positions reflect the infant’s postural control abilities through a series of temporal and spatial changes of CoP that can be analyzed using posturometric parameters. The reliability of the new PT for the recognition of postural control disturbances in preterm high-risk infants was determined by comparing its outcomes between group of pre-term infants with normal FMs (prognosis of a normal future motor outcome) and pre-term infants who presented with abnormal FMs (a high risk of later neurological dysfunction). Significant differences were found for both types of posturometric indices based on CoP shifts, i.e., the velocity of the CoP displacement (Vmean CoP; VmaxCoP) and the sway path of the CoP (SPL) and the linear displacement of the CoP in left, right, up, and down (MaxCoPR; MaxCoPL; MaxCoPup; MaxCoPdown) (Table 3), as well as posturometric indices based on the surface area of the CoP (ACoP, MDevCoP, MCoPx, and MCoPy) in the supine position (Table 3) and posturometric indices based on the surface area of the CoP (ACoP, MDevCoP, MCoPx, and MCoPy) in the prone position (Table 4), and these results partially confirmed our first hypothesis. The results showed that new PT in the supine position had the ability to distinguish preterm infants who presented with normal FMs, i.e normally developing infants, from those who presented with abnormal FMs, i.e., infants at risk for neuromotor deficits. The obtained results supported our previous finding [19,20] that PT in the supine position can be a particularly revealing indicator for the development of postural control abnormalities in preterm infants.

Due to the fact that there have been no studies on the reliability of PT based on the displacement of CoP on the BoS in a horizontal position in infants (except for our last study), it is difficult to compare our results with those found in previous studies. So far, only a few studies have included the recognition of postural control of infants using CoP methodology [14,15,16,17,18]. These studies used PSM to measure CoP displacement [14,15,16,17,18,28,29]. In addition, several studies have shown that CoP displacements derived from PSM were less reliable compared with those measured with force plates [14,18].

Recently, the CoP methodology with force plates was used by Kyvelidouet et al. and Harbourne et al. to analyze infant movements [28,29]. While they examined measures of CoP displacement in typically developing infants and infants at risk for CP, they included infants who were over one year old and assessed their sitting postural control. However, the recognition of potential postural control disturbances is especially important in the first few months of life when infants set the foundations of their future motor ability. Our findings demonstrate the suitability of the proposed early validation of CoP displacement in infants (up to 3 months of age) measured using a force platform in a horizontal position (supine) to distinguish normally developing infants from infants at risk for neuromotor deficits.

The current study also aimed to evaluate the repeatability of the new PT based on a CoP displacement analysis in a repeated measures design. The relative test–retest analysis showed that PT in both supine and prone positions has excellent and good repeatability for all variables based on the surface area of CoP and minimum moderate repeatability for all variables based on CoP displacement (Table 5 and Table 6). In addition, as we assumed, PT in both positions displayed higher repeatability in the group of infants who presented with abnormal FMs than in the group of infants who presented with normal FMs (Table 5 and Table 6). This finding means that the obtained results confirmed our second hypothesis about high repeatability of the new PT. This is probably in line with the different nature of GM patterns presented by these two different groups of pre-term infants. Normal and abnormal GMs significantly vary in many kinematic parameters, such as sequence, speed, and amplitude. These differences concern normal and abnormal FMs. Normal FMs are intensive small movements of moderate speed with variable acceleration of the neck, trunk, and limbs in all directions, while abnormal GMs, such as poor repertoire GMs or cramped synchronized GMs, have a monotonous sequence of movements and a reduced variance in speed and amplitude of movements and are characterized by a habit pattern [4,5]. Considering the strong relationship between postural and motor control, the higher repeatability of outcomes using PT in infants with abnormal FMs seems justified. This finding is also confirmed by the general significant correlation between all outcomes of PT in supine and prone positions in infants with abnormal FMs and the lack of this relationship in infants with normal FMs. These findings supported our last hypothesis that PT will display better reliability and test–retest repeatability in infants with abnormal FMs than in infants with normal FMs.

To summarize, it can be said that the new PT in supine position based on the measurement of CoP displacement allows to distinguish normally developing infants from infants at risk for neuromotor deficits (up to 3 months of age). Additionally, the high test–retest repeatability of PT means that the design and reliability of the new PT are appropriate. The new PT represents an important toward objective, sensor-supported infant postural control assessment.

### Limitations

This study has some limitations. First, the number of participants in our study was relatively small; however, the low prevalence of CP (and thus abnormal GMs), which remains at 2–3 per 1000 live births, was a significant limitation in recruiting a greater number of infants with consistent abnormal GMs in the experimental group.

Second, PT was performed only in the “fidgety period” i.e., around 3 months of age. Follow-up longitudinal studies are needed to provide some evidence that pre-term infants with normal GMs and normal postural control will normally develop and these infants who presented with abnormal GMs and altered postural parameters will develop postural and motor development disorders.

## 5. Conclusions

Although the current study yielded promising results, further longitudinal research in preterm infants should identify whether altered postural control parameters prognose future motor outcomes, i.e., normal future motor outcomes and typically developing infants vs. infants with or at risk of developing NDI and later neurological dysfunction.

## Figures and Tables

**Table 1:** Characteristics of the groups of infants with normal FMs and infants with abnormal FMs.

Parameter	Normal FMs (*n* = 18)		Abnormal FMs (*n* = 19)	
	Mean (SD)	Min–Max	Mean (SD)	Min–Max
Birth weight (grams)	975.83 (273.41)	690–1850	949.68 (209.50)	650–1430
Gestational age (weeks)	27.72 (2.16)	24–32	27.15 (1.50)	25–30
Apgar at fifth minute (score)	6.33 (2.40)	1–9	5.31 (1.67)	3–8
Neonatal Medical Index (NMI) (score)	2.1 (0.9)	1–5	3.6 (1.6)	1–5
Length of time stay in hospital (days)	27 (16)	2–65	31 (20)	5–85
Delivery, normal n (%)	8 (44)		6 (32)	
Caesarean, n (%)	10 (56)		13 (68)	
Sex, girls, n (%)	6 (33)		6 (32)	
Boys, n (%)	12 (67)		13 (68)	

**Table 2:** Posturometric indices based on CoP shifts and posturometric indices based on the surface area of the CoP during lying.

**Posturometric Indices Based on CoP Shifts During Lying**	
Vmax CoP	Maximal velocity of the CoP displacement [cm/s]
Vmean CoP	Mean velocity of the CoP displacement [cm/s]
SPL	Sway path length of the CoP [cm]
MaxCoPR	Maximal linear displacement of the CoP in a medial–lateral direction (in right side) [cm]
MaxCoPL	Maximal linear displacement of the CoP in a medial–lateral direction (in left side) [cm]
MaxCoPup	Maximal linear displacement of the CoP in an anterior–posterior direction (up) [cm]
MaxCoPdown	Maximal linear displacement of the CoP in an anterior–posterior sway path of the CoP (down) [cm]
**Posturometric Indices Based on the Area of the CoP During Lying**	
ACoP	The area of CoP shifts under the unrolled trajectory [cm^2^] is $ACoP=\overset{N}{\underset{i=2}{\sum }}p\left(i\right)\;$ where: $p\left(i\right)=\sqrt{ob\left(i\right)\cdot \left[ob\left(i\right)-r\left(i-1\right)\right]\cdot \left[ob\left(i\right)-r\left(i\right)\right]\cdot \left[ob\left(i\right)-l\left(i\right)\right]}$ *p*(*i*) is the surface area of a triangle comprised of two successive points of a given trajectory (*T_c_*(*i* − 1), *T_c_*(*i*)); *T_c_*_0_ represents the center of that trajectory; and *l*(*i*) is given by the formula, whereas the values of *r*(*i*), *r*(*i* − 1), and *ob* (*i*) are calculated using the following equations: $r\left(i-1\right)=\sqrt{{\left[x_{c}\left(i-1\right)-x_{c0}\right]}^{2}+{\left[y_{c}\left(i-1\right)-y_{c0}\right]}^{2}}$ $ob\left(i\right)=\frac{l\left(i\right)+r\left(i\right)+r\left(i-1\right)}{2}$
MDevCoP	Mean CoP deviation from the center of the trajectory [cm] is $MDevCoP=\frac{\sum_{i=1}^{N}r\left(i\right)}{N}\;\;\;$ where: *N*—number of points comprising the trajectory, *r*(*i*) distance of the *i*-th point of the trajectory from its center, and *T_c_*_0_ is given by the formula.
MCoPx	Mean medial–lateral linear displacement of the CoP [cm] $MCoPx=\frac{\sum_{i=1}^{N}\left|x_{C}\left(i\right)-X_{CO}\right|}{N}$
MCoPy	Mean posterior–anterior displacement of the CoP [cm] $MCoPy=\frac{\sum_{i=1}^{N}\left|y_{C}\left(i\right)-Y_{CO}\right|}{N}$

**Table 3:** Differences in CoP parameters describing the spontaneous sway of CoP and the area of CoP between the groups of infants who presented with normal and abnormal FMs in PT in the supine position.

Parameter	Normal FMs (*n* = 18)	Abnormal FMs (*n* = 19)	Statistical Test, *p*-Value
	M (SD)	M (SD)	
Vmean CoP [cm/s]	7.09 (36.57)	3.64 (21.35)	*t* (37) = −3.34, *p* = 0.01
VmaxCoP [cm/s]	8.78 (33.18)	5.88 (30.05)	*t* (37) = −2.71, *p* = 0.01
SPL [cm]	26.34 (9.95)	17.65 (9.02)	*t* (37) = −2.71, *p* = 0.01
MaxCoPR [cm]	1.15 (0.40)	0.63 (0.32)	*t* (37) = −4.20, *p* < 0.01
MaxCoPL [cm]	2.71 (0.81)	2.00 (1.08)	*t* (37) = −2.23, *p* = 0.03
MaxCoPup [cm]	3.46 (1.14)	1.97 (0.96)	*t* (37) = −4.14, *p* < 0.01
MCoPdown [cm]	4.06 (1.83)	2.12 (1.19)	*t* (37) = −3.64, *p* < 0.01
AcoP [cm^2^]	37.61 (13.49)	20.30 (9.48)	*t* (37) = −4.34, *p* < 0.01
MdevCoP [cm]	33.87 (12.68)	18.63 (9.93)	*t* (37) = −3.93, *p* < 0.01
McoPx [cm]	0.74 (0.28)	0.40 (0.23)	*t* (37) = −3.75, *p* < 0.01
McoPy [cm]	0.71 (0.31)	0.39 (0.20)	*t* (37) = −3.51, *p* < 0.01

Statistical test; student’s *t*-test (*t*; *p*).

**Table 4:** Differences in CoP parameters describing the spontaneous sway of CoP and the area of CoP between the groups of infants who presented with normal and abnormal FMs in PT in the prone position.

Parameter	Normal FMs (*n* = 18)	Abnormal FMs (*n* = 19)	Statistical Test, *p*-Value
	M (SD)	M (SD)	
Vmean CoP [cm/s]	6.27 (2.59)	5.70 (2.22)	*t* (37) = 0.70, *p* = 0.49
VmaxCoP [cm/s]	10.65 (34.06)	8.26 (3.99)	*t* (37) = −1.93, *p* = 0.06
SPL [cm]	31.95 (10.21)	24.80 (11.99)	*t* (37) = −1.93, *p* = 0.06
MaxCoPR [cm]	3.53 (1.40)	2.26 (1.19)	*t* (37) = −2.94, *p* = 0.07
MaxCoPLl [cm]	3.57 (1.43)	2.67 (1.36)	*t* (37) = −1.93, *p* = 0.06
MaxCoPup [cm]	4.21 (1.90)	2.93 (1.23)	*t* (37) = −2.33, *p* = 0.06
MCoPdown [cm]	3.20 (0.94)	3.05 (1.07)	*t* (37) = 0.44, *p* = 0.66
ACoP [cm^2^]	37.33 (14.23)	25.63 (11.60)	*t* (37) = −2.67, *p* = 0.01
MDevCoP [cm]	1.24 (0.46)	0.88 (0.36)	*t* (37) = −2.52, *p* = 0.02
MCoPx [cm]	0.84 (0.39)	0.57 (0.27)	*t* (37) = −2.31, *p* = 0.03
MCoPy [cm]	0.74 (0.28)	0.55 (0.24)	*t* (37) = −2.10, *p* = 0.04

Statistical test; student’s *t*-test (*t*; *p*).

**Table 5:** Intraclass correlation coefficients for the first and second measurements of individual parameters describing the spontaneous sway of CoP and the area of CoP in the whole group of infants, i.e., those who presented with normal and abnormal FMs patterns in PT in supine and prone positions.

Parameter	Supine Position (*n* = 37)			Prone Position (*n* = 37)		
	ICC	95% CI		ICC	95% CI	
Vmean CoP [cm/s]	0.83	0.69	0.91	0.77	0.59	0.87
VmaxCoP [cm/s]	0.61	0.36	0.78	0.47	0.17	0.69
SPL [cm]	0.83	0.69	0.91	0.77	0.59	0.87
MaxCoPR [cm]	0.59	0.34	0.77	0.41	0.11	0.65
MaxCoPLl [cm]	0.57	0.31	0.76	0.51	0.22	0.72
MaxCoPup [cm]	0.73	0.53	0.85	0.51	0.30	0.75
MCoPdown [cm]	0.62	0.38	0.79	0.41	0.09	0.65
ACoP [cm^2^]	0.92	0.85	0.96	0.91	0.84	0.96
MDevCoP [cm]	0.91	0.83	0.95	0.90	0.81	0.95
MCoPx [cm]	0.84	0.71	0.91	0.72	0.52	0.85
MCoPy [cm]	0.85	0.73	0.92	0.89	0.80	0.94

**Table 6:** Intraclass correlation coefficients for the first and second measurements of individual parameters describing the spontaneous sway of CoP and the area of CoP in the groups of infants who presented with normal and abnormal FMs in PT in prone and supine positions.

Parameter	Supine Position						Prone Position					
	Normal FMs (*n* = 18)			Abnormal FMs (*n* = 19)			Normal FMs (*n* = 18)			Abnormal FMs (*n* = 19)		
	ICC	95% CI		ICC	95% CI		ICC	95% CI		ICC	95% CI	
Vmean CoP [cm/s]	0.93	0.89	0.95	0.82	0.74	0.88	0.75	0.58	0.85	0.83	0.69	0.91
VmaxCoP [cm/s]	0.52	0.34	0.66	0.80	0.70	0.86	0.49	0.23	0.69	0.61	0.36	0.78
SPL [cm]	0.93	0.89	0.95	0.94	0.88	0.97	0.79	0.64	0.88	0.82	0.67	0.90
MaxCoPR [cm]	0.59	0.43	0.72	0.52	0.34	0.66	0.57	0.33	0.74	0.56	0.30	0.75
MaxCoPLl [cm]	0.55	0.38	0.68	0.52	0.27	0.71	0.52	0.27	0.71	0.56	0.29	0.74
MaxCoPup [cm]	0.62	0.46	0.73	0.80	0.70	0.86	0.58	0.34	0.75	0.66	0.44	0.81
MCoPdown [cm]	0.52	0.34	0.66	0.61	0.36	0.78	0.47	0.21	0.67	0.61	0.36	0.78
ACoP [cm^2^]	0.82	0.74	0.88	0.90	0.83	0.95	0.90	0.83	0.95	0.91	0.83	0.95
MDevCoP [cm]	0.90	0.85	0.93	0.97	0.94	0.98	0.89	0.81	0.94	0.90	0.81	0.94
MCoPx [cm]	0.81	0.73	0.88	0.88	0.79	0.93	0.76	0.61	0.86	0.84	0.71	0.91
MCoPy [cm]	0.87	0.80	0.91	0.84	0.71	0.91	0.88	0.79	0.93	0.84	0.71	0.91

**Table 7:** Pearson’s correlations between CoP parameters in PT in prone and supine positions in the groups of infants with normal versus abnormal FMs.

Parameter	Normal FMs	Abnormal FMs
Vmean CoP [cm/s]	r = 0.15, *p* = 0.52	r = 0.65, *p* < 0.01
VmaxCoP [cm/s]	r = 0.04, *p* = 0.85	r = 0.51, *p* = 0.04
SPL [cm]	r = 0.04, *p* = 0.85	r = 0.71, *p* < 0.01
MaxCoPR [cm]	r = −0.18, *p* = 0.45	r = 0.36, *p* = 0.17
MaxCoPLl [cm]	r = −0.01, *p* = 0.97	r = 0.49, *p* = 0.05
MaxCoPup [cm]	r = 0.09, *p* = 0.71	r = 0.41, *p* = 0.12
MCoPdown [cm]	r = −0.05, *p* = 0.85	r = 0.63, *p* < 0.01
ACoP [cm^2^]	r = −0.32, *p* = 0.16	r = 0.73, *p* < 0.01
MDevCoP [cm]	r = −0.16, *p* = 0.49	r = 0.72, *p* < 0.01
MCoPx [cm]	r = −0.14, *p* = 0.55	r = 0.69. *p* < 0.01
MCoPy [cm]	r = −0.37, *p* = 0.10	r = 0.72, *p* < 0.01

## Data Availability

The data supporting the results of this study are available from the corresponding author upon reasonable request from any qualified investigator.

## References

[1] Bosanquet M., Copeland L., Ware R., Boyd R. (2013). A systematic review of tests to predict cerebral palsy in young children. Dev. Med. Child Neurol..

[2] Novak I., Morgan C., Adde L., Blackman J., Boyd R.N., Brunstrom-Hernandez J., Cioni G., Damiano D., Darrah J., Eliasson A.C. (2017). Early, Accurate Diagnosis and Early Intervention in Cerebral Palsy: Advances in Diagnosis and Treatment. JAMA Pediatr..

[3] Caesar R., Colditz P.B., Cioni G., Boyd R.N. (2021). Clinical tools used in young infants born very preterm to predict motor and cognitive delay (not cerebral palsy): A systematic review. Dev. Med. Child Neurol..

[4] Einspieler C., Prechtl H.F. (2005). Prechtl’s assessment of general movements: A diagnostic tool for the functional assessment of the young nervous system. Ment. Retard. Dev. Disabil. Res. Rev..

[5] Prechtl H.F., Einspieler C., Cioni G., Bos A.F., Ferrari F., Sontheimer D. (1997). An early marker for neurological deficits after perinatal brain lesions. Lancet.

[6] Pires C.D.S., Marba S.T.M., Caldas J.P.S., Stopiglia M.C.S. (2020). Predictive value of the general movements assessment in preterm infants: A meta-analysis. Rev. Paul. Pediatr..

[7] Robinson H., Hart D., Vollmer B. (2021). Predictive validity of a qualitative and quantitative Prechtl’s General Movements Assessment at term age: Comparison between preterm infants and term infants with HIE. Early Hum. Dev..

[8] Chen H., Xue M., Mei Z., Bambang Oetomo S., Chen W. (2016). A Review of Wearable Sensor Systems for Monitoring Body Movements of Neonates. Sensors.

[9] Adde L., Helbostad J.L., Jensenius A.R., Taraldsen G., Grunewaldt K.H., Støen R. (2010). Early prediction of cerebral palsy by computer-based video analysis of general movements: A feasibility study. Dev. Med. Child Neurol..

[10] Adde L., Helbostad J., Jensenius A.R., Langaas M., Støen R. (2013). Identification of fidgety movements and prediction of CP by the use of computer-based video analysis is more accurate when based on two video recordings. Physiother. Theory Pract..

[11] Adde L., Yang H., Sæther R., Jensenius A.R., Ihlen E., Cao J.Y., Støen R. (2018). Characteristics of general movements in preterm infants assessed by computer-based video analysis. Physiother. Theory Pract..

[12] Støen R., Songstad N.T., Silberg I.E., Fjørtoft T., Jensenius A.R., Adde L. (2017). Computer-based video analysis identifies infants with absence of fidgety movements. Pediatr. Res..

[13] Marcroft C., Khan A., Embleton N.D., Trenell M., Plötz T. (2015). Movement recognition technology as a method of assessing spontaneous general movements in high risk infants. Front. Neurol..

[14] Dusing S.C., Kyvelidou A., Mercer V.S., Stergiou N. (2009). Infants born preterm exhibit different patterns of center-of-pressure movement than infants born at full term. Phys. Ther..

[15] Dusing S.C., Izzo T.A., Thacker L.R., Galloway J.C. (2014). Postural complexity differs between infant born full term and preterm during the development of early behaviors. Early Hum. Dev..

[16] Prosser L.A., Aguirre M.O., Zhao S., Bogen D.K., Pierce S.R., Nilan K.A., Zhang H., Shofer F.S., Johnson M.J. (2022). Infants at risk for physical disability may be identified by measures of postural control in supine. Pediatr. Res..

[17] Rihar A., Sgandurra G., Beani E., Cecchi F., Pašič J., Cioni G., Dario P., Mihelj M., Munih M. (2016). CareToy: Stimulation and Assessment of Preterm Infant’s Activity Using a Novel Sensorized System. Ann. Biomed. Eng..

[18] Martinez-Cesteros J., Medrano-Sanchez C., Plaza-Garcia I., Igual-Catalan R., Albiol-Pérez S.A. (2021). Velostat-Based Pressure-Sensitive Mat for Center-of-Pressure Measurements: A Preliminary Study. Int. J. Environ. Res. Public Health.

[19] Kniaziew-Gomoluch K., Szopa A., Łosień T., Kidoń Z., Domagalska-Szopa M. (2022). Postural stability of children born prematurely in the perinatal risk group. Pol. J. Physiother..

[20] Kniaziew-Gomoluch K., Szopa A., Kidoń Z., Siwiec A., Domagalska-Szopa M. (2023). Design and construct validity of a postural control test for pre-term infants. Diagnostics.

[21] Einspieler C., Prechtl H.F.R., Bos A.F., Ferrari F., Cioni G. (2008). Prechtl’s Method on the Qualitative Assessment of General Movements in Preterm, Term and Young Infants.

[22] Łukaszewicz T., Kania D., Kidoń Z., Pethe-Kania K. (2015). Posturographic methods for body posture symmetry assessment. Bull. Pol. Acad. Sci. Tech. Sci..

[23] Faul F., Erdfelder E., Lang A.G., Buchner A. (2007). G*Power 3: A flexible statistical power analysis program for the social, behavioral, and biomedical sciences. Behav. Res. Methods.

[24] Faul F., Erdfelder E., Buchner A., Lang A.G. (2009). Statistical power analyses using G*Power 3.1: Tests for correlation and regression analyses. Behav. Res. Methods.

[25] Portney L.G., Watkins M.P. (2000). Foundations of Clinical Research: Applications to Practice.

[26] Altman D.G. (2018). Practical Statistics for Medical Research.

[27] Koo T.K., Li M.Y. (2016). A Guideline of Selecting and Reporting Intraclass Correlation Coefficients for Reliability Research. J. Chiropr. Med..

[28] Kyvelidou A., Harbourne R.T., Shostrom V.K., Stergiou N. (2010). Reliability of center of pressure measures for assessing the development of sitting postural control in infants with or at risk of cerebral palsy. Arch. Phys. Med. Rehabil..

[29] Harbourne R.T., Deffeyes J.E., Kyvelidou A., Stergiou N. (2009). Complexity of postural control in infants: Linear and nonlinear features revealed by principal component analysis. Nonlinear Dyn. Psychol. Life Sci..

